# Context matters: How do task demands modulate the recruitment of sensorimotor information during language processing?

**DOI:** 10.3389/fnhum.2022.976954

**Published:** 2023-01-17

**Authors:** Emiko J. Muraki, Alison Doyle, Andrea B. Protzner, Penny M. Pexman

**Affiliations:** ^1^Department of Psychology, University of Calgary, Calgary, AB, Canada; ^2^Hotchkiss Brain Institute, University of Calgary, Calgary, AB, Canada

**Keywords:** embodied cognition, semantic representation, EEG, ERP, body-object interaction

## Abstract

Many theories of semantic representation propose that simulations of sensorimotor experience contribute to language processing. This can be seen in the body-object interaction effect (BOI; how easily the human body can interact with a word’s referent). Words with high BOI ratings (e.g., *ball*) are processed more quickly than words with low BOI ratings (e.g., *cloud*) in various language tasks. This effect can be modulated by task demands. Previous research established that when asked to decide if a word is an object (entity condition), a BOI effect is observed, but when asked to decide if a word is an action (action condition), there is no BOI effect. It is unclear whether the null behavioral effect in the action condition reflects top-down modulation of task-relevant sensorimotor information or the absence of bottom-up activation of sensorimotor simulations. We investigated this question using EEG. In Experiment 1 we replicated the previous behavioral findings. In Experiment 2, 50 participants were assigned to either the entity or action conditions and responded to the same word stimuli. In both conditions we observed differences in ERP components related to the BOI effect. In the entity condition the P2 mean amplitude was significantly more positive for high compared to low BOI words. In the action condition the N400 peak latency was significantly later for high compared to low BOI words. Our findings suggest that BOI information is generated bottom-up regardless of task demands and modulated by top-down processes that recruit sensorimotor information relevant to the task decision.

## Introduction

In recent years theories of semantic representation and processing have diverged in the role proposed for embodied or sensorimotor information. These theories fall on a theoretical spectrum ranging from those proposing no relationship between semantic representation and sensorimotor systems to more embodied theories which propose that semantic meaning is entirely dependent on sensorimotor systems (Meteyard et al., 2012). Much evidence has converged on a middle ground between these two extremes, supporting hybrid or multidimensional accounts of semantic representation that propose word meaning relies on both sensorimotor and language-specific representations. Although there is general support for these accounts, there remain several questions as to the dynamics of multidimensional semantic processing. Traditional accounts of semantic memory assumed that semantic representation is relatively fixed and, at least at core, context-free (e.g., Collins and Loftus, 1975). An advantage of hybrid or multidimensional accounts of embodied semantic representation is that they afford flexibility in semantic processing such that it might be influenced by contextual demands or individual differences (Barsalou, 2020), with some proponents going as far as stating that a concept is never represented the same way twice (Connell and Lynott, 2014).

There are still several unanswered questions regarding how semantic representation and processing are influenced by context. In some cases, context is thought to influence the extent to which sensorimotor simulations are activated during semantic processing. Situations that require deeper semantic processing might activate simulations to a greater extent than tasks that require only surface-level processing, for which more invariant linguistic representations are thought to suffice (Barsalou et al., 2008; Dove, 2016). Another view is that concepts can never be separated from their context (Yee and Thompson-Schill, 2016). A concept is not one singular representation but rather a group of conceptual representations distributed across multiple brain regions, and not every representation needs to be recruited to successfully derive meaning. Only context-relevant representations need be accessed, and thus access may be modulated *via* either top-down selection of context-relevant sensorimotor simulations or bottom-up activation of context-relevant sensorimotor simulations.

Behavioral studies have found that the recruitment of sensorimotor information during language processing can be modulated by whether the task demands deep (i.e., semantic) vs. shallow (i.e., linguistic) processing. Mega-studies of language processing have found that sensorimotor effects are either not present in lexical processing tasks (e.g., lexical decision) or tend to be weaker than sensorimotor effects observed in semantic processing tasks (Yap et al., 2012; Goh et al., 2016; Pexman et al., 2019). However, there is also evidence that when tasks require deeper semantic processing, only context-relevant sensorimotor information is recruited. For instance, motor regions are activated when processing action verbs in literal contexts but not non-literal contexts (Raposo et al., 2009; Schuil et al., 2013; Yang and Shu, 2016), object nouns are processed more quickly when a semantic context emphasizes the functional use of the object and the response movement is congruent with the direction of the functional use (Van Dam et al., 2010), verbs are recalled more accurately when participants imagine themselves, rather than a non-human robot, completing an action (Sidhu and Pexman, 2016), and action word processing is associated with increased grip force when a sentence emphasizes an agent’s action and decreased grip force when the action word is negated (Aravena et al., 2012, 2014). These findings indicate that the recruitment of sensorimotor information is flexible, allowing for the optimal strategy to be adopted given the task demands.

There is also evidence that neural activity related to semantic processing can be modulated by task demands. West and Holcomb (2000) observed concreteness effects in both response time (faster responses to concrete words) and event related potentials (ERP; more negative N400 when processing concrete words) when participants made a semantic decision, but not when they made a surface level decision, such as identifying a specific letter within a word. Another way to manipulate context is by changing the task demands, which can modulate the type of simulated sensorimotor information that is recruited. Motor and auditory brain regions are differentially activated depending on whether the task requires action or sound judgment (Popp et al., 2019; Kuhnke et al., 2020). Words associated with both action and color have been associated with increased neural activity in either action areas (e.g., posterior intraparietal sulcus) or visual processing regions (e.g., fusiform gyrus) depending on whether the task emphasized color or action properties of the word (van Dam et al., 2012). In some cases, neural differences are observed when behavioral differences are not evident. Hargreaves et al. (2012b) manipulated the task demands for a semantic decision (*Is it an animal?* and *Is it a concrete thing?*) using the same word stimuli in both conditions. While no significant differences between conditions were observed in the participants’ behavioral data, they found greater BOLD response in brain regions associated with knowledge of living things (e.g., the fusiform and inferior temporal gyrus) in the animal condition and more in the motor cortex in the concrete condition. This suggests that the nature of the sensorimotor simulations that were recruited was task dependent.

In the present study we investigated the neural correlates of a task-dependent sensorimotor effect: the body-object interaction effect. Body-object interaction (BOI) is a measure which indexes how easily the human body can interact with a word’s referent (Siakaluk et al., 2008a). For example, *ball* is a high BOI word, presumably because it has many different possible bodily interactions (e.g., catching, throwing, etc.), whereas the word *cloud* is a low BOI word as it refers to something with which it is more difficult for a human body to interact. In lexical-semantic processing tasks a BOI effect is consistently observed, wherein reaction times are faster to high BOI words than low BOI words (Siakaluk et al., 2008a,b; Tillotson et al., 2008; Pexman et al., 2019). This suggests that high BOI words benefit from sensorimotor simulations that contribute to the semantic processing of these words. Tousignant and Pexman (2012) showed that simply altering the task instructions given to participants appeared to modulate whether BOI information was recruited to categorize words. They compared semantic decision reaction times and accuracy for words that were high and low in BOI. When the semantic decision was framed as *“entity or non-entity”* they observed the expected BOI effect. In contrast, when the decision was *“action or non-action”*, no BOI effect was observed.

The findings from Tousignant and Pexman (2012) suggest that BOI information is only recruited when it is relevant to task demands, which is inconsistent with a strong embodiment view of semantic processing, but behavioral findings do not provide clear insight on the neural mechanisms involved in recruiting this information. For example, in the action condition, recruitment of BOI information might be modulated in a top-down manner, by ignoring sensorimotor simulations that are automatically engaged during semantic processing. In contrast, BOI information may not be activated at all in the action condition, indicating bottom-up modulation of semantic processing with sensorimotor simulations only engaged when that information is relevant to the task decision. We investigated these possibilities through the use of electroencephalography (EEG), examining whether task demands modulated ERP components associated with sensorimotor effects in language processing (the P2 and the N400).

The P2 is a positive ERP component that occurs approximately 150–250 ms after stimulus onset (peaking at ∼200 ms) and is typically located over centro-frontal regions. The P2 is modality independent, meaning that it occurs for visual, auditory, and somatosensory stimuli (Crowley and Colrain, 2004). A larger P2 component has been associated with processing words with more semantic information (e.g., number of features; Kounios et al., 2009; Rabovsky et al., 2012) and with semantic context (Federmeier and Kutas, 2002; Baccino and Manunta, 2005; Barber et al., 2011). Other studies have found sensorimotor effects, with a larger P2 amplitude at central electrodes for action words than non-action words (Pérez-Gay Juárez et al., 2019). There is also evidence of context modulating P2 sensorimotor effects. Amsel et al. (2013) found that task demands modulated the time course of sensorimotor activity, with living/non-living vs. graspable/ungraspable decisions eliciting a more positive P2, suggesting that living thing knowledge is recruited earlier than action representations. van Dam et al. (2014) observed an interaction between task context and response direction on P2 amplitude, with a larger P2 amplitude when context emphasized the functional use of an object (e.g., *thirst*-cup) and the response movement was incongruent with the functional use of an object (e.g., respond by moving the hand away from the body, rather than toward the body like when drinking from a cup). Xue et al. (2015) observed a marginal effect of context, wherein low BOI words in semantically rich contexts had a more positive P2 than low BOI words in semantically poor contexts.

The N400 component is a negative ERP waveform that typically begins 200 ms after stimulus onset and lasts approximately 300 ms (peaking at ∼400 ms; Swaab et al., 2012), usually occurring over centro-parietal areas. The classic N400 was associated with semantic incongruence (e.g., the sentence *The dog jumped the fence* elicits a smaller N400 than *The dog jumped the turtle*; Kutas and Hillyard, 1980), however, it has since been associated more broadly with semantic processing (Kutas and Federmeier, 2011). Words associated with more semantic information have shown both smaller (Kounios et al., 2009; Taler et al., 2013, 2016) and larger (Müller et al., 2010; Laszlo and Federmeier, 2011; Rabovsky et al., 2012) N400 amplitudes compared to words associated with relatively less semantic information. There have also been sensorimotor effects observed in the N400, again with mixed results. An N400 concreteness effect has been observed, with a larger N400 associated with processing concrete concepts (Amsel and Cree, 2013; Barber et al., 2013; Dalla Volta et al., 2014), yet other studies have not found a concreteness effect in the N400 (Muraki et al., 2020). Xue et al. (2015) identified a context-dependent effect, with a weaker N400 for high BOI words that were presented in semantically rich contexts compared to semantically poor contexts. However, for low BOI words there was less difference in the N400 between contexts, suggesting they do not benefit from context to the same extent as high BOI words. Finally, Al-Azary et al. (2022) found that low-BOI words evoked a larger N400 than high-BOI words did, but only when the task context emphasized the touchability of a word’s referent. Thus, while the N400 is sometimes sensitive to semantic processing and sensorimotor effects, the mechanisms underlying these effects are not well understood.

In Experiment 1 we conducted a replication of the Tousignant and Pexman (2012) study to first determine whether the same behavioral effects were observed despite any behavioral changes caused by the change in testing environment (i.e., the EEG recording chamber). Then, in Experiment 2, we examined the associated ERP components using a slightly modified procedure. The change in procedure was a delayed participant response designed to eliminate a motor response that could produce a Lateralized Readiness Potential (LRP; Müller and Hagoort, 2006; Killikelly and Szűcs, 2013) and other motor-related potentials (MRPs; van Vliet et al., 2014) which could contaminate the P2 and N400 components. In both experiments the task decisions were identical to the most extreme conditions in Tousignant and Pexman (2012). That is, two groups of participants were asked to categorize the same set of words based on the decision condition to which they were assigned: an entity condition (*Is it an entity or a non-entity?*) vs. an action condition (*Is it an action or a non-action?*).

If we observe differences in the ERP components between high and low BOI words in only the entity condition, this would indicate that BOI information is activated *via* bottom-up processes only when sensorimotor simulations are relevant to the task. Alternatively, if we observe differences in the ERP components between high and low BOI words in both conditions, this would suggest that BOI information is activated and then modulated *via* a top-down process, and that sensorimotor simulations are generated regardless of task demands. To allow for better understanding of the null effect that we hypothesized may occur in the action condition, we investigated differences in both mean amplitude and peak latency in the P2 and N400 components. Based on P2 and N400 literature, in the current study we hypothesized that the P2 component would be more positive for high BOI words compared to low BOI words but made no directional predictions about whether high BOI words would be associated with smaller or larger N400 amplitudes, or earlier or later peak latencies in either the P2 or N400 compared to low BOI words.

## Experiment 1

### Materials and methods

#### Participants

We recruited 40 participants who participated in exchange for monetary compensation ($30 for community participants) or bonus credit in a Psychology undergraduate course (student participants). All participants reported being healthy, right-handed, native English speakers with no history of severe brain injury. Three participants were excluded from analysis, one due to behavioral performance no greater than chance and two due to issues with data acquisition. Following these exclusions, the analysis included 37 participants (7 males). Participants were pseudo randomly assigned to conditions: 19 participants (3 males) in the “Entity” condition (“Is it an entity or a non-entity?”; *M* age = 21.37, *SD* = 2.45) and 18 participants (4 males) in the “Action” condition (“Is it an action or a non-action?”; *M* age = 20.44, *SD* = 2.06).

#### Stimuli

Stimuli for this experiment were adapted from words used in Tousignant and Pexman (2012). To increase the number of observations per word type to that appropriate for EEG analysis we selected an additional 60 words (15 High BOI, 15 Low BOI, and 30 action words) making the final stimuli set 200 words (50 High BOI, 50 Low BOI, and 100 action words). To evaluate the suitability of these additional stimuli, a separate group of 31 University of Calgary undergraduate participants rated how “action like” each of 300 potential stimuli were using a six-point Likert scale (1 = entity, 6 = action). We then used these ratings to select the final stimuli list of 200 words. Mean ratings for high and low BOI words can be found in Table 1. As with the Tousignant and Pexman study, the high and low BOI words were matched on several lexical and semantic dimensions to ensure the words were as similar as possible in all regards except for BOI (see Table 1). Additionally, the high and low BOI words were matched with the action words for length and frequency to ensure the length and frequency of the words would not cue the participants to make a particular categorization response.

**TABLE 1:** Mean (SD) characteristics of low body-object interaction (BOI), high body-object interaction (BOI), and action word stimuli.

Characteristic	Low BOI words	High BOI words	Low vs. High BOI (*p*-value)	Action words
BOI	3.00 (0.77)	5.59 (0.44)	<0.001	n/a
Action-like	1.42 (0.24)	1.48 (0.31)	0.31	n/a
Word length	4.42 (1.05)	4.30 (0.84)	0.53	4.35 (0.85)
Imageability	570.28 (70.07)	577.12 (59.39)	0.60	n/a
Concreteness	564.80 (47.15)	570.96 (47.14)	0.52	n/a
Orthographic neighbors	7.80 (5.93)	8.58 (5.91)	0.51	7.27 (5.27)
Log HAL frequency	9.24 (1.59)	9.24 (1.27)	0.97	9.56 (1.64)

SD, standard deviation; BOI, body-object interaction. BOI ratings were taken from Tillotson et al. (2008) norms. Imageability and concreteness ratings were taken from the MRC psycholinguistic database (Wilson, 1988), orthographic neighbors and log HAL frequency measures were taken from the English lexicon project (Balota et al., 2007).

#### Procedure

Participants sat in a comfortable chair in a sound attenuated chamber. In the semantic decision task words were presented one at a time on a computer screen using Presentation^®^ software (Version 18.3, Neurobehavioral Systems, Inc., Albany, CA, USA^1^). Participants were asked to categorize each word based on the condition to which they had been assigned (“Is it an action or a non-action?” for the action condition and “Is it an entity or a non-entity?” for the entity condition). The keys “D” and “K” were used on a computer keyboard to indicate responses with the “K” key indicating the “yes” response for each condition. Participants were first given 20 practice trials with feedback on the accuracy of their categorization. Upon completion of the practice trials participants completed the 200 experimental trials. The order of word presentation was randomized for each participant.

For each trial, participants saw a central fixation cross for 500 ms followed by blank screen for a variable duration between 0–500 ms and finally the onset of the stimulus. Participants then provided their semantic decision as to whether the word referred to an action or entity dependent on their assigned task condition. The stimuli remained on the screen until the participant made a response, triggering a 1,000 ms blank screen before the onset of the next trial.

#### Data acquisition

Continuous EEG was recorded in a dimly lit, electrically shielded, soundproof chamber using an EasyCap (10/20 positioning system) containing 64 electrodes (using Cz as the reference). Our focus in the present experiment was on the behavioral data, but we did collect EEG data and pre-processed and analyzed those data in the same manner as described for Experiment 2, below. EEG results for Experiment 1 are presented as Supplementary material for the interested reader, but not presented or interpreted in the main text due to concerns about effects of the immediate response paradigm on ERP components.

### Results

Any words for which participants demonstrated less than 60% categorization accuracy were removed from the analysis. This included *back*, *knit*, *smile*, *song*, *speck*, *stripe*, and *well* in the entity condition (3.5% of the data) and *cope* in the action condition (0.5% of the data). Trials with response times faster than 200 ms or slower than 3,000 ms were also removed from the analysis (entity condition: 0.53% of the data; action condition: 0.0% of the data). Finally, response times that were more than three standard deviations below or above an individual participant’s mean were removed (entity condition: 1.87% of the data; action condition: 1.80% of the data).

To determine if task demands modulated the effect of BOI on mean reaction time or accuracy we conducted the same analyses as in Tousignant and Pexman (2012): 2 (condition: Action/Entity) × 2 (BOI: High/Low BOI) mixed factors ANOVAs by subjects (F1) and by items (F2). Means and standard deviations for each word type and condition are reported in Table 2. In the subject-wise analysis, condition was the between-subject variable and BOI was the within-subject variable. In the item-wise analysis, condition was the within-item variable and BOI was the between-item variable.

**TABLE 2:** Mean (SD) response time and accuracy by word type and task condition in experiment 1.

Word type	Entity condition		Action condition	
	RT (ms)	Accuracy	RT (ms)	Accuracy
High BOI	852 (166)	0.96 (0.05)	747 (107)	0.95 (0.04)
Low BOI	915 (200)	0.90 (0.07)	763 (97)	0.95 (0.04)

SD, standard deviation; BOI, body-object interaction; RT, response time; ms, milliseconds.

#### Reaction time

A significant interaction was observed for condition and BOI, *F*1(1, 35) = 5.26, *p* = 0.03, η^2^ = 0.13; *F*2(1, 93) = 11.18, *p* = 0.001, η^2^ = 0.11. In the entity condition high BOI words were categorized faster than low BOI words, *t*1(18) = 3.48, *p* = 0.003; *t*2(44) = 3.34, *p* = 0.002, but no significant difference was found in the reaction times for high BOI and low BOI words in the action condition, *t*1(17) = 1.57, *p* = 0.14; *t*2(49) = 1.01, *p* = 0.32. The results also included a main effect of BOI *F*1(1, 35) = 14.06, *p* = 0.001, η^2^ = 0.29; *F*2(1, 93) = 6.34, *p* = 0.01, η^2^ = 0.06, as responses were faster for high BOI words than for low BOI words overall.

#### Accuracy rates

A significant interaction was observed for condition and BOI, *F*(1, 35) = 5.67, *p* = 0.02, η^2^ = 0.14; *F*2(1, 93) = 8.63, *p* = 0.004, η^2^ = 0.09. In the entity condition responses were more accurate for high BOI words than low BOI words, *t*1(18) = 3.12, *p* = 0.01; *t*2(44) = 3.16, *p* = 0.003. No significant difference was observed between high and low BOI accuracy rates in the action condition *t*1(17) = 0.41 *p* = 0.69; *t*2(49) = 0.30, *p* = 0.76. The results also included a main effect of BOI *F*(1, 35) = 7.91, *p* = 0.01, η^2^ = 0.18; *F*2(1, 93) = 3.99, *p* = 0.04, η^2^ = 0.04, as responses were more accurate for high BOI words than for low BOI words overall.

### Discussion

The behavioral results from Experiment 1 replicate those reported in Tousignant and Pexman (2012). That is, when given the same words under different task demands (i.e., decision framed as either entity vs. non-entity or action vs. non-action) participants only appeared to process BOI information in the entity condition and not in the action condition. This replication indicates that the pattern of results reported by Tousignant and Pexman persists when participants are placed in a sound attenuated chamber, have an electrode cap placed on their head, and when the item set used in Tousignant and Pexman is expanded considerably. These behavioral results provide evidence that participants recruited different information in each condition, suggesting that the recruitment of semantic information is a dynamic process dependent on task demands. This is consistent with results of previous studies that manipulated semantic task demands (Hargreaves et al., 2012b; Yap et al., 2012). We also observed a significant difference in the P2 amplitude between high and low BOI words in the entity condition, but not the action condition (reported in the Supplementary material). However, we have not interpreted this difference due to concerns that the immediate response in our study design response might influence the ERP results, through the LRP and MRP.

## Experiment 2

A limitation of Experiment 1 was that immediate behavioral responses were collected on all trials, to replicate as many aspects of the procedure used in Tousignant and Pexman (2012) as possible. This may have contaminated the ERP results with the LRP and other MRPs. As the LRP component occurs over the motor cortex, it overlaps with components from electrodes in that area (Smulders and Miller, 2012). The LRP also has a negative potential causing it to increase potentials of negative components and decrease potentials of positive components. Thus, in Experiment 1 the LRP could have contaminated the EEG data by increasing the N400 waveforms and decreasing the P2 waveforms. Previous literature has also shown that requiring participants to respond as quickly as possible to stimuli produces MRPs that overlap with the N400 and can lead to misinterpretation (van Vliet et al., 2014). To address this issue, we utilized a 2,000 ms delayed response in Experiment 2. The duration of the delay was chosen based on reaction times from Experiment 1 and delayed responses used in previous literature (van Elk et al., 2010; Madan et al., 2016). This paradigm should ensure that any MRP would occur long after the participant has fully processed the word.

### Materials and methods

#### Participants

We recruited 55 participants who participated in exchange for monetary compensation ($40 for community participants) or bonus credit in a Psychology undergraduate course (student participants). All participants reported being healthy, right-handed, native English speakers with no history of severe brain injury. Ten participants were excluded from analysis, two due to behavioral performance no greater than chance, two due to issues with data acquisition, one due to falling outside our specified age range for participants (18–35), and five for excessively noisy ERP data. Following these exclusions, the analysis included data for 45 participants (29 females). Participants were pseudo-randomly assigned to conditions: 22 participants (14 females) in the “Entity” condition (“Is it an entity or a non-entity?”; *M* age = 22.05, *SD* = 2.72) and 23 participants (15 females) in the “Action” condition (“Is it an action or a non-action?”; *M* age = 21.26, *SD* = 3.02).

#### Stimuli

The stimuli were identical to those used in Experiment 1.

#### Procedure

The procedure for this experiment was identical to that of Experiment 1 except for two aspects of the response. In this experiment a delayed response was implemented in which the participant could not respond until after 2,000 ms. This interval was 500 ms longer than the slowest responses in Experiment 1. The stimulus remained on screen for 2,000 ms. At this point the words “yes” and “no” appeared below the word. The appearance of these words indicated the participant could now respond and reminded the participant which response each key indicated. For example, if the “K” key indicated a yes response the word “Yes” would appear on the right. The assignment of the response keys was counterbalanced between participants with the “K” key denoting a yes response for half of participants in each condition and the “D” key denoting a yes response for the other half in each condition.

#### Data acquisition and pre-processing

The data recording was identical to that of Experiment 1. The Brain Vision antiCHamp system with active electrodes (Brain Products GmbH) was used and impedances were kept below 17 kOhms for the duration of the recording. The reference electrode was Cz and raw data were filtered at 0.1–55 Hz. Noisy channels were interpolated before being re-referenced to an average reference to exclude the excessive noise from the common average. The data were segmented into epochs ranging from 200 ms pre-stimulus onset to 1,000 ms post-stimulus onset. All epochs were baseline corrected using the first 200 ms portion of the epoch. Trials in which a stimulus onset was followed by a correct response were binned according to word type (e.g., high BOI, low BOI, and action). Using the program EEGLAB (Delorme and Makeig, 2004) artifact removal was performed through independent components analysis (ICA) and components consisting of artifacts such as eye blinks, saccades, horizontal eye movements, or other muscle artifacts were removed.

### Results

#### Behavioral results

In Experiment 2 there were two words in the low entity condition (*core* and *well*) for which participants were accurate 59% of the time, falling just under the threshold of 60% categorization accuracy that we used in Experiment 1. However, to retain as many correct trials as possible for calculating the ERP components, we left these two words in the analysis. The data from Experiment 2 were analyzed the same way as in Experiment 1, except that in this instance only response accuracy was analyzed (with the delayed response paradigm response latencies were not interpretable). Accuracy means and standard deviations for each word type and condition are reported in Table 3.

**TABLE 3:** Mean (SD) response accuracy by word type and task condition in experiment 2.

Word type	Entity condition	Action condition
High BOI	0.96 (0.05)	0.96 (0.04)
Low BOI	0.89 (0.12)	0.97 (0.03)

SD, standard deviation; BOI, body-object interaction.

#### Accuracy rates

A significant interaction was observed for condition and BOI, *F*1(1, 43) = 11.68, *p* = 0.001, η^2^ = 0.21; *F*2(1, 98) = 12.85, *p* < 0.001, η^2^ = 0.12. In the entity condition responses were more accurate for high BOI words than low BOI words, *t*1(21) = 4.07, *p* < 0.001; *t*2(49) = 3.59, *p* < 0.001. No significant difference was observed between high and low BOI accuracy rates in the action condition *t*1(22) = −0.67, *p* = 0.479; *t*2(49) = −1.00, *p* = 0.314. We also observed a significant main effect of BOI, *F*1(1,43) = 5.85, *p* = 0.020, η^2^ = 0.12; *F*2(1,98) = 6.26, *p* = 0.014, η^2^ = 0.06 and a significant main effect of condition, *F*1(1,43) = 4.75, *p* = 0.035, η^2^ = 0.12; *F*2(1,98) = 14.96, *p* < 0.001, η^2^ = 0.13.

#### ERP results

**FIGURE 1:**
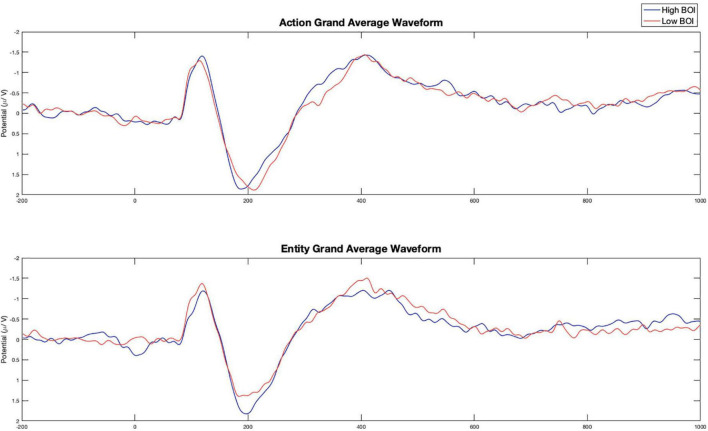
Experiment 2 grand averaged event related potentials (ERPs) across all electrodes by task condition and word type. Grand average was calculated from a pre-defined central cluster of electrodes (FC1, FCz, FC2, C1, Cz, C2, CP1, CPz, and CP2).

**FIGURE 2:**
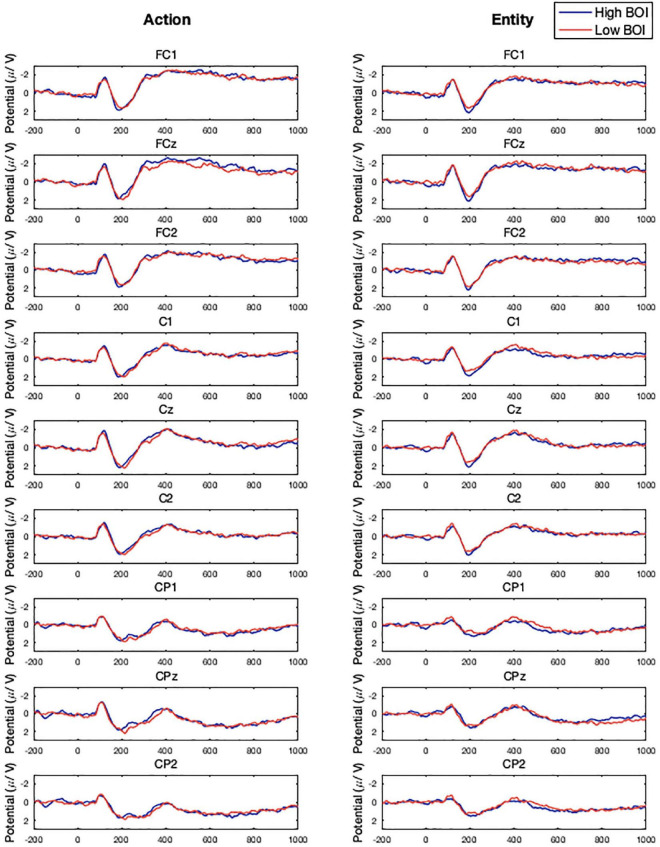
Experiment 2 grand averaged, electrode-specific event related potentials (ERPs) by task condition and word type.

The P2 and N400 ERPs were extracted using ERPLAB (Lopez-Calderon and Luck, 2014) from a pre-defined central cluster of electrodes (FC1, FCz, FC2, C1, Cz, C2, CP1, CPz, CP2) that are consistent with the localized sources of BOI effects identified in previous fMRI studies (Hargreaves et al., 2012a). Mean amplitude of the P2 was extracted for three 40 ms time windows (140–180, 180–220, and 220–260 ms) and for the N400 using three 50 ms time windows (350–400, 400–450, and 450–500). We assessed differences in P2 and N400 mean amplitude using 3 (Time window) × 2 (Word Type) × 2 (Task Condition) ANOVAs. Peak latency was analyzed using a 140–260 ms time window and 350–500 ms time window for the P2 and N400 components, respectively. We assessed differences in P2 and N400 peak latency using 2 (Word Type) × 2 (Task Condition) ANOVAs. Planned *a priori* paired samples *t*-tests were used to compare the P2 and N400 ERP components for word types within each task condition. The ERPs representing the average across these electrodes in the action and entity conditions are presented in Figure 1 and the ERPs for all nine electrodes included in our analyses are presented in Figure 2.

### P2

#### Mean amplitude

A 3 (Time window; 140–180 ms vs. 180–220 ms vs. 220–260 ms) × 2 (Word Type; High BOI vs. Low BOI) × 2 (Task Condition; Entity vs. Action) ANOVA was conducted. Mauchly’s Test indicated a violation of sphericity for the main effect of time window [χ^2^(2) = 27.37, *p* < 0.001] and for the interactions with time window [χ^2^(2) = 13.08, *p* = 0.001], so the degrees of freedom were corrected using the Greenhouse Geiser method (ε = 0.68 and ε = 0.79 for the main effect and interactions, respectively). Levene’s test indicated no violation of homogeneity of variance. The three-way interaction between time window, word type, and task condition was not significant, *F*(1.58, 67.85) = 2.04, *p* = 0.148, partial η^2^ = 0.05. There were also no significant interactions between time window and word type, *F*(1.58, 67.85) = 1.63, *p* = 0.208, partial η^2^ = 0.04, time window and task condition, *F*(1.35, 58.16) = 0.03, *p* = 0.930, partial η^2^ = 0.001, or word type and task condition, *F*(1,43) = 1.35, *p* = 0.251, partial η^2^ = 0.03. There was a significant main effect of time window, *F*(1.35, 58.16) = 15.30, *p* < 0.001, partial η^2^ = 0.26, but no significant main effect of word type, *F*(1,43) = 0.06, *p* = 0.816, partial η^2^ = 0.001 or task condition, *F*(1,43) = 0.47, *p* = 0.497, partial η^2^ = 0.01. Pairwise comparisons between time windows revealed a significantly less positive mean amplitude between 140–180 ms (*M* = 0.69, *SD* = 0.22) compared to between 180–220 ms (*M* = 1.61, *SD* = 0.18), with *p* < 0.001. There was also a difference between 180–220 ms and 220–260 ms (*M* = 1.02, *SD* = 0.16) with significantly more positive mean amplitude between 180–200 ms, *p* < 0.001.

Planned comparisons revealed a significant difference in mean amplitude between 180–220 ms post-stimulus onset in the entity condition. High BOI words (*M* = 1.67, *SD* = 1.23) had a significantly more positive mean amplitude than low BOI words (*M* = 1.34, *SD* = 0.99), *t*(21) = 2.51, *p* = 0.020. No other planned comparisons were significant. See Table 4 for all planned comparison tests.

**TABLE 4:** P2 mean amplitude, standard deviations, and *t*-tests by task condition, word type, and time window.

Time window	Entity condition			Action condition		
	High BOI M(SD)	Low BOI M(SD)	*t*	High BOI M(SD)	Low BOI M(SD)	*t*
140–180ms	0.57 (1.57)	0.55 (1.37)	0.09	0.85 (1.55)	0.80 (1.51)	0.31
180–220ms	1.67 (1.23)	1.34 (0.99)	2.51*	1.68 (1.23)	1.76 (1.31)	−0.43
220–260ms	0.97 (1.10)	0.88 (1.02)	0.42	0.98 (1.25)	1.25 (1.43)	−1.33

M, mean; SD, standard deviation. *Indicates *p* < 0.05.

#### Peak latency

A 2 (Word Type; High BOI vs Low BOI) × 2 (Task Condition; Entity vs. Action) ANOVA was conducted. Mauchly’s Test indicated no violation of sphericity and Levene’s test indicated no violation of homogeneity of variance. The interaction between word type and task condition was not significant, *F*(1,43) = 0.09, *p* = 0.930, partial η^2^ = 0.00. The main effects of word type, *F*(1,43) = 0.58, *p* = 0.451, partial η^2^ = 0.01, and task condition, *F*(1,43) = 0.23, *p* = 0.634, partial η^2^ = 0.01, were also not significant. Planned comparisons revealed no significant differences in P2 peak latency for high and low BOI words in either task condition. See Table 5 for all planned comparison tests.

**TABLE 5:** P2 mean peak latency, standard deviations, and *t*-tests by task condition and word type.

Entity condition			Action condition		
High BOI M(SD)	Low BOI M(SD)	*t*	High BOI M(SD)	Low BOI M(SD)	*t*
198.64 (18.92)	200.91 (23.53)	−0.47	201.48 (24.67)	204.35 (30.15)	−0.61

M, mean; SD, standard deviation.

### N400

#### Mean amplitude

A 3 (Time window; 350–400 ms vs. 400–450 ms vs. 450–500 ms) × 2 (Word Type; High BOI vs. Low BOI) × 2 (Task Condition; Entity vs. Action) ANOVA was conducted. Mauchly’s Test indicated a violation of sphericity for the main effect of time window [χ^2^(2) = 24.63, *p* < 0.001], so the degrees of freedom were corrected using the Greenhouse Geiser method (ε = 0.69). Levene’s test indicated no violation of homogeneity of variance. The three-way interaction between time window, word type, and task condition was not significant, *F*(2, 86) = 0.43, *p* = 0.653, partial η^2^ = 0.01. There were no significant interactions between time window and word type, *F*(2, 86) = 0.60, *p* = 0.552, partial η^2^ = 0.01, time window and task condition, *F*(2, 86) = 0.12, *p* = 0.884, partial η^2^ = 0.003, or word type and task condition, *F*(1,43) = 0.47, *p* = 0.678, partial η^2^ = 0.004. There was a significant main effect of time window, *F*(1.39, 59.57) = 6.16, *p* = 0.009, partial η^2^ = 0.13, but no significant main effect of word type, *F*(1,43) = 0.17, *p* = 0.678, partial η^2^ = 0.004 or task condition, *F*(1,43) = 0.002, *p* = 0.965, partial η^2^ = 0.000. Pairwise comparisons between time windows revealed a significantly more negative mean amplitude between 350–400 ms (*M* = −1.14, *SD* = 0.21) and 400–450 ms (*M* = −1.21, *SD* = 0.18) compared to between 450–500 ms (*M* = −0.86, *SD* = 0.18), with *p* = 0.037 and *p* < 0.001, respectively. There was no significant difference between 350–400 ms and 400–450 ms, *p* = 0.537. Planned comparisons revealed no significant differences in mean amplitude between high and low BOI words in either the action or entity conditions. See Table 6 for all planned comparison tests.

**TABLE 6:** N400 mean amplitude, standard deviations, and *t*-tests by task condition, word type, and time window.

Time window	Entity condition			Action condition		
	High BOI M(SD)	Low BOI M(SD)	*t*	High BOI M(SD)	Low BOI M(SD)	*t*
350–400 ms	−1.09 (1.64)	−1.22 (1.51)	0.84	−1.21(1.12)	−1.05 (1.49)	−0.86
400–450 ms	−1.12 (1.33)	−1.27 (1.28)	0.75	−1.23 (1.09)	−1.23 (1.43)	−0.01
450–500 ms	−0.81 (1.33)	−0.97 (1.17)	0.69	−0.81 (1.14)	−0.84 (1.46)	0.18

M, mean; SD, standard deviation.

#### Peak latency

A 2 (Word Type; High BOI vs. Low BOI) × 2 (Task Condition; Entity vs. Action) ANOVA was conducted. Mauchly’s Test indicated no violation of sphericity and Levene’s test indicated no violation of homogeneity of variance. The interaction between word type and task condition was not significant, *F*(1,43) = 2.07, *p* = 0.157, partial η^2^ = 0.05. The main effects of word type, *F*(1,43) = 3.67, *p* = 0.062, partial η^2^ = 0.08, and task condition, *F*(1,43) = 0.62, *p* = 0.435, partial η^2^ = 0.01, were also not significant. Planned comparisons revealed a significant difference in N400 peak latency between high BOI words (*M* = 441.13, *SD* = 52.31) and low BOI words (*M* = 411.13, *SD* = 53.66), in the action task condition, *t*(22) = 2.94, *p* = 0.008. No other significant differences were identified in the planned comparisons. See Table 7 for all planned comparison tests.

**TABLE 7:** N400 mean peak latency, standard deviations, and *t*-tests by task condition and word type.

Entity condition			Action condition		
High BOI M(SD)	Low BOI M(SD)	*t*	High BOI M(SD)	Low BOI M(SD)	*t*
438.00 (44.58)	433.73 (53.30)	0.29	441.13 (52.31)	411.14 (53.66)	2.94*

M, mean; SD, standard deviation. *Indicates *p* < 0.05.

## General discussion

The purpose of the present study was to understand the neural mechanisms associated with a behavioral, task-dependent, body-object interaction effect. In Experiment 1 we confirmed that the task-dependent BOI effect identified by Tousignant and Pexman (2012) could be replicated in an EEG recording environment and with additional stimuli. In Experiment 2 we examined differences between task conditions in the P2 and N400 amplitude and latency. We hypothesized that if differences in neural activity for high and low BOI words were observed only in the entity condition, this would indicate that the task-dependent BOI effect is driven by bottom-up mechanisms which selectively activate a simulation of sensorimotor experience if it is relevant to the task decision. Alternatively, if differences in neural activity were observed in both the entity and the action conditions, this would indicate that the task-dependent BOI effect is driven by top-down mechanisms which selectively attend to sensorimotor simulations only when they are relevant to the task decision. Our results do not fit perfectly with either account, and suggest that both bottom-up and top-down processes are involved in the BOI effect: we observed differences in ERP components in both conditions, but these differences emerged in different components (P2 and N400) and different measures (mean amplitude and peak latency).

In the entity condition the P2 mean amplitude was significantly more positive for high compared to low BOI words (an effect also observed in Experiment 1 but not interpreted due to concerns of motor contamination in the ERP component due to the immediate participant responses). This finding is consistent with previous research findings that processing more semantic information, such as words whose referents have more features, action words with more embodied meaning, or low BOI words in semantically rich contexts, are associated with a larger P2 component (Kounios et al., 2009; Rabovsky et al., 2012; Xue et al., 2015; Pérez-Gay Juárez et al., 2019). This finding is also consistent with those of van Dam et al. (2014), who found that incongruency between actual movement and movement implied by a context led to a larger P2 component, suggesting that action representations were accessed and could interfere with task performance. The timing of the P2 component indicates that BOI information is engaged early in semantic processing and is therefore likely to reflect access of relevant sensorimotor simulations as part of concept retrieval (Kiefer et al., 2022), rather than cascading activation from the concept to sensorimotor regions associated with that concept (Mahon and Caramazza, 2008).

Our findings in the action condition were less clear. In the action condition we observed no differences in N400 mean amplitude (consistent with the findings of Al-Azary et al., 2022), however, the N400 peak latency was significantly later for high compared to low BOI words. This would suggest that BOI information is generated bottom-up regardless of task demands and modulated by top-down processes that recruit sensorimotor information relevant to the task decision, otherwise there should be no difference between high and low BOI words in the action condition. However, the interpretation of N400 peak latency differences is challenging, as one of the hallmarks of the N400 component it that its latency is remarkably stable (Kutas and Federmeier, 2011). There is some limited evidence that N400 latency may be related to delayed semantic processing and integration. Aphasics with mild comprehension deficits show a later N400 peak latency when processing sentences (Khachatryan et al., 2017) and there is an earlier N400 peak latency for primed or related relative to unprimed or unrelated words (Deacon et al., 1995; Soto and França, 2020; Tiedt et al., 2020).

One potential explanation for the N400 peak latency difference observed in the action condition is that motor representations of object interactions that are activated for high BOI words may interfere with making an action or non-action task decision. Previous research has found that BOI can facilitate or inhibit processing speed and accuracy, depending on whether the task decision was framed as “*Is the word concrete?*” vs. “*Is the word abstract?*”, respectively (Newcombe et al., 2012). This raises an interesting question: if sensorimotor simulations are initiated regardless of task condition, what is the mechanism for selectively attending to task relevant information and does task irrelevant information require some form of suppression? The controlled semantic cognition framework (Lambon Ralph et al., 2017) proposes that a control network interacts with the semantic representation network, to selectively focus on task or context relevant semantic information, while suppressing competitors or strong associations. However, it’s unclear whether the control network would also be responsible for suppressing task irrelevant information related to the same concept, rather than a competitor or associate. The lack of difference in the action condition between P2 components for high and low BOI words suggests that the mechanism for selectively attending to task relevant information is engaged early in semantic processing. It is also possible that differences in the N400 peak latency reflect cascading activation of BOI information accessed after task-relevant semantic information has been accessed. Of course, our interpretations of the N400 peak latency difference are speculative and will require systematic study in future research.

One interpretation of the present findings could be that semantic representations are fixed, as high BOI words appear to activate sensorimotor simulations regardless of task demands. However, we observed no differences in the P2 component in our action condition, which reflects early stages of semantic processing. This suggests that sensorimotor simulations are not activated in the same manner in both the entity and action condition. Therefore, we believe our findings are more consistent with the proposal of context-sensitivity from Yee and Thompson-Schill (2016) and provide further insight into the mechanisms by which context-relevant representations are accessed, through top-down selection of relevant sensorimotor simulations. Our findings are also consistent with hybrid or multidimensional accounts of semantic representation. Sensorimotor simulations appear to be activated in both task conditions, but this information does not influence semantic processing when it is not relevant to the task demands. Therefore, there must be other (non-sensorimotor) semantic information that is attended to when sensorimotor information is not relevant to the task.

Our findings give insight into the mechanisms used to access context-relevant information during semantic processing, however, it is unclear whether these same mechanisms would be engaged in adjustments to semantic processing that are observed in tasks using more shallow semantic processing, such as that used in a lexical decision task. Therefore, future research might consider whether a similar top-down modulation is employed in tasks that require only shallow processing of a word. This research would further refine our understanding of conceptual flexibility, by testing whether the mechanisms for attending to context-relevant information may themselves vary depending on the nature of the task.

Future research might also extend our findings to the investigation of abstract concept representation and processing. The present stimuli were highly concrete words, but there is substantial evidence that abstract concepts are also associated with sensorimotor information (Connell and Lynott, 2012, 2014; Dreyer et al., 2015; Dreyer and Pulvermuller, 2018; Harpaintner et al., 2018, 2020). Furthermore, some accounts of semantic representation propose that the dichotomy between concrete and abstract concepts is no longer a meaningful distinction, as concepts are always processed in relation to a situation that may highlight different dimensions of word meaning that are relevant to the context (Barsalou, 2020). Therefore, it would be informative to examine whether the top-down processes that we observed in the present study are also engaged in selecting context-relevant information when processing abstract words, and in which contexts sensorimotor dimensions of abstract word meaning are considered relevant to semantic processing.

There are some limitations to consider when drawing conclusions from our results. First, we did not systematically investigate whether individual differences such as gender or motor expertise (e.g., athlete or dancer) might be related to the effects we observed in this study, and there is evidence of individual differences being related to sensorimotor effects in language processing (Ibanez et al., 2022) and in body-object interaction effects in particular (Muraki and Pexman, 2021). Therefore, some caution should be used when considering the generalizability of these findings.

An additional limitation is the potential influence of individual items within our stimuli set. Although we carefully matched our word stimuli on lexical-semantic variables known to influence word processing (e.g., length, frequency, orthographic neighbors, concreteness), the high and low BOI stimuli still vary on these dimensions. Unfortunately, due to the nature of our measure of neural activity (ERP), and the way the data were collected, we are not able to analyze trial-level data to account for random effects of word, nor are we able to conduct item-wise analyses on the ERP data. Item-level analyses of the behavioral data in Experiment 1 did suggest that the effects remain the same whether the analyses are conducted by subject or by item. Nonetheless, analyzing the data using a mixed-effects approach would allow for the random effects of participant (e.g., learning and fatigue) and word (e.g., order of presentation, item characteristics) to be accounted for Baayen et al. (2008).

Finally, although the ROIs we identified in our ERP analyses were based on the neural correlates of BOI effects observed in previous research (Hargreaves et al., 2012a), the spatial resolution of EEG is poor, therefore we cannot state with certainty that the P2 component differences observed in the entity condition and the N400 differences observed in the action condition correspond to the same neural generator as that identified in Hargreaves et al. Future research using fMRI may provide additional confirmation that the task-dependent effects we observed are consistent with neural regions related to kinesthetic memory such as the left inferior parietal lobule. Such research may also provide additional insight on the neural regions associated with the top-down modulation and selection of context-relevant information.

In conclusion, the present results provide new insight into the mechanisms of context-dependent semantic processing. Our findings suggest that BOI information is generated bottom-up regardless of task demands, and that task demands modulate top-down processes that recruit sensorimotor information relevant to the task decision. This supports the proposal that concept representation is multidimensional, and that conceptual processing is flexible rather than invariant. This is also consistent with more recent theories of semantic cognition (Barsalou, 2020) that propose that conceptual representation and processing is situated and influenced by external states such as the environment and context.

### Open practices statement

The data and materials for the experiments reported here are available upon request, and none of the experiments were pre-registered.

## Data availability statement

The raw data supporting the conclusions of this article will be made available by the authors, without undue reservation.

## Ethics statement

The studies involving human participants were reviewed and approved by the University of Calgary Conjoint Faculties Research Ethics Board. The patients/participants provided their written informed consent to participate in this study.

## Author contributions

AD, AP, and PP contributed to the conception and design of the study. AD collected the data. EM performed the statistical analysis. EM and AD wrote the first draft of the manuscript. All authors contributed to the manuscript revision, read, and approved the submitted version.

## Footnotes

1
www.neurobs.com
