# Supplementary material for: Context matters: How do task demands modulate the recruitment of sensorimotor information during language processing?
Source: Front Hum Neurosci. 2023 Jan 17;16:976954. doi: 10.3389/fnhum.2022.976954 (PMC9886877; doi:10.3389/fnhum.2022.976954)

Supplementary Material

# Experiment 1 ERP Results

The P2 and N400 ERPs were extracted using ERPLAB (Lopez-Calderon & Luck, 2014) from a pre-defined central cluster of electrodes (FC1, FCz, FC2, C1, Cz, C2, CP1, CPz, CP2) that are consistent with the localized sources of BOI effects identified in fMRI work (Hargreaves et al., 2012). Mean amplitude of the P2 was extracted for three 40 ms time windows (140-180, 180-220, and 220-260 ms) and for the N400 using three 50 ms time windows (350-400, 400-450, and 450-500). We assessed differences in P2 and N400 mean amplitude using 3 (Time window) x 2 (Word Type) x 2 (Task Condition) ANOVAs. Peak latency was analyzed using 140-260 ms and 350-500 ms time windows for the P2 and N400 components respectively. We assessed differences in P2 and N400 peak latency using 2 (Word Type) x 2 (Task Condition) ANOVAs. Planned a priori paired samples t-tests were used to compare the P2 and N400 ERP components for word types within each task condition. The ERPs representing the average across these electrodes in the action and entity conditions are presented in Figure 1 and the ERPs for all 9 electrodes included in our analyses are presented in Figure 2.

## P2 Mean Amplitude

A 3 (Time window; 140 – 180ms vs 180 – 220ms vs 220 – 260ms) x 2 (Word Type; High BOI vs Low BOI) x 2 (Task Condition; Entity vs Action) ANOVA was conducted. Mauchly’s Test indicated a violation of sphericity for the main effect of time window [χ^2^(2) = 9.67, *p* = .008] and for the interactions with time window [χ^2^(2) = 17.60, *p* < .001], so the degrees of freedom were corrected using the Greenhouse Geiser method (ε = 0.80 and ε = 0.71 for the main effect and interactions respectively). Levene’s test indicated a violation of homogeneity of variance between groups in the third time window (220 – 260 ms) for both high and low BOI words, however ANOVA is robust to such violations. The three-way interaction between time window, word type, and task condition was not significant, *F*(1.42, 49.86) = 0.50, *p* = .546, partial η^2^ = 0.01. There were also no significant interactions between time window and word type, *F*(1.42, 49.86) = 0.42, *p* = .592, partial η^2^ = 0.01, time window and task condition, *F*(1.60, 56.11) = 2.37, *p* = .113, partial η^2^ = 0.06, or word type and task condition, *F*(1,35) = 0.87, *p* = .357, partial η^2^ = 0.02. There was a significant main effect of time window, *F*(1.60, 56.11) = 9.54, *p* < .001, partial η^2^ = 0.21, but no significant main effect of word type, *F*(1,35) = 3.95, *p* = .055, partial η^2^ = 0.10 or task condition, *F*(1,35) = 0.10, *p* = .749, partial η^2^ = 0.003. Pairwise comparisons between time windows revealed a significantly less positive mean amplitude between 140 – 180ms (*M* = 0.13, *SD* = 1.36) compared to between 180 – 220ms (*M* = 1.07, *SD* = 0.18) and 220 – 260ms (*M* = 0.73, *SD* = 0.22) with *p* < .001 and *p* = .032 respectively.

Planned comparisons revealed a significant difference in mean amplitude between 180 – 220ms post-stimulus onset in the entity condition. High BOI words (*M* = 1.19, *SD* = 1.10) had a significantly more positive mean amplitude than low BOI words (*M* = 0.70, *SD* = 1.24), *t*(18) = 2.70, *p* = .015. No other planned comparisons were significant. See Table 1 for all planned comparison tests.

## P2 Peak Latency

A 2 (Word Type; High BOI vs Low BOI) x 2 (Task Condition; Entity vs Action) ANOVA was conducted. Levene’s test indicated no violation of homogeneity of variance. The interaction between word type and task condition was not significant, *F*(1,35) = 0.32, *p* = .575, partial η^2^ = 0.009. The main effects of word type, *F*(1,35) = 0.29, *p* = .594, partial η^2^ = 0.01, and task condition, *F*(1,35) = 3.72, *p* = .062, partial η^2^ = 0.10, were also not significant. Planned comparisons revealed no significant differences in P2 peak latency for high and low BOI words in either task condition. See Table 2 for all planned comparison tests.

## N400 Mean Amplitude

A 3 (Time window; 350 – 400ms vs 400 – 450ms vs 450 – 500ms) x 2 (Word Type; High BOI vs Low BOI) x 2 (Task Condition; Entity vs Action) ANOVA was conducted. Mauchly’s Test indicated a violation of sphericity for the main effect of time window [(χ^2^(2) = 21.75, *p* < .001], so the degrees of freedom were corrected using the Greenhouse Geiser method (ε = 0.68). Levene’s test indicated a violation of homogeneity of variance between groups for all time windows but 400 – 450 ms in the low BOI word type, however ANOVA is robust to this violation. The three-way interaction between time window, word type, and task condition was not significant, *F*(1.97, 68.87) = 0.16, *p* = .848, partial η^2^ = 0.01. There were no significant interactions between time window and task condition, *F*(1.36, 47.54) = 0.88, *p* = .384, partial η^2^ = 0.03, or word type and task condition, *F*(1,35) = 0.25, *p* = .622, partial η^2^ = 0.01. However, there was a significant interaction between time window and word type, *F*(1.97, 68.87) = 4.380, *p* = .017, partial η^2^ = 0.11. A significant main effect of time window was observed, *F*(1.36, 47.54) = 8.32, *p* = .001, partial η^2^ = 0.19, but no significant main effect of word type, *F*(1,35) = 0.49, *p* = .488, partial η^2^ = 0.014 or task condition, *F*(1,35) = 0.962, *p* = .333, partial η^2^ = 0.03.

Follow-up simple main effects found that for high BOI words there was a significant difference between the third time window (450 – 500ms; *M* = -0.68, *SD* = 1.77) compared to both the first (350 – 400ms; *M* = -1.35, *SD* = 1.75) and second time windows (400 – 450ms; *M* = -1.41, *SD* = 1.84), with *p* = .001 and *p* < .001 respectively. For low BOI words, there was a significant difference between the second window (400 – 450ms; *M* = -1.34, *SD* = 1.82) and third window (450 – 500ms; *M* = -0.97, *SD* = 1.91), *p* = .007. Planned comparisons revealed no significant differences in mean amplitude between high and low BOI words in either the action or entity conditions. See Table 3 for all planned comparison tests.

## N400 Peak Latency

A 2 (Word Type; High BOI vs Low BOI) x 2 (Task Condition; Entity vs Action) ANOVA was conducted. Mauchly’s Test indicated no violation of sphericity and Levene’s test indicated no violation of homogeneity of variance. The interaction between word type and task condition was not significant, *F*(1,35) = 0.08, *p* = .781, partial η^2^ = 0.002. The main effects of word type, *F*(1,35) = 0.05, *p* = .832, partial η^2^ = 0.001, and task condition, *F*(1,35) = 0.02, *p* = .897, partial η^2^ = 0.000, were also not significant. Planned comparisons revealed no significant differences in N400 peak latency between high and low BOI words in either task condition. See Table 4 for all planned comparison tests.

# Supplementary Tables

## Supplementary Table 1

*P2 Mean Amplitude, Standard Deviations, and T-tests by Task Condition, Word Type, and Time Window*

| Time Window | Entity Condition | | | Action Condition | | |
| --- | --- | --- | --- | --- | --- | --- |
|  | High BOI *M(SD)* | Low BOI *M(SD)* | *t* | High BOI *M(SD)* | Low BOI *M(SD)* | *t* |
| 140 – 180ms | 0.07 (1.08) | -0.25 (1.02) | 1.47 | 0.43 (0.77) | 0.27 (0.83) | 0.88 |
| 180 – 220ms | 1.19 (1.10) | 0.70 (1.24) | 2.70* | 1.26 (0.97) | 1.15 (1.26) | 0.58 |
| 220 – 260ms | 1.09 (0.67) | 0.83 (1.05) | 1.50 | 0.55 (1.85) | 0.44 (1.81) | 0.44 |

*Note.* M = Mean, SD = Standard Deviation. * indicates *p* < .05.

## Supplementary Table 2

*P2 Mean Peak Latency, Standard Deviations, and T-tests by Task Condition and Word Type*

| Entity Condition | | | Action Condition | | |
| --- | --- | --- | --- | --- | --- |
| High BOI *M(SD)* | Low BOI *M(SD)* | *t* | High BOI *M(SD)* | Low BOI *M(SD)* | *t* |
| 212.32 (29.59) | 216.63 (28.57) | -0.61 | 199.33 (23.19) | 199.22 (24.78) | 0.04 |

*Note.* M = Mean, SD = Standard Deviation.

## Supplementary Table 3

*N400 Mean Amplitude, Standard Deviations, and T-tests by Task Condition, Word Type, and Time Window*

| Time Window | Entity Condition | | | Action Condition | | |
| --- | --- | --- | --- | --- | --- | --- |
|  | High BOI *M(SD)* | Low BOI *M(SD)* | *t* | High BOI *M(SD)* | Low BOI *M(SD)* | *t* |
| 350 – 400ms | -0.96 (1.12) | -1.09 (0.89) | 0.61 | -1.77 (2.19) | -1.80 (1.90) | 0.14 |
| 400 – 450ms | -1.17 (1.15) | -1.15 (1.46) | -0.11 | -1.67 (2.38) | -1.55 (2.17) | -0.47 |
| 450 – 500ms | -0.45 (1.20) | -0.85 (1.27) | 1.91 | -0.92 (2.24) | -1.10 (2.45) | 0.80 |

*Note.* M = Mean, SD = Standard Deviation.

## Supplementary Table 4

*N400 Mean Peak Latency, Standard Deviations, and T-tests by Task Condition and Word Type*

| Entity Condition | | | Action Condition | | |
| --- | --- | --- | --- | --- | --- |
| High BOI *M(SD)* | Low BOI *M(SD)* | *t* | High BOI *M(SD)* | Low BOI *M(SD)* | *t* |
| 439.47 (55.52) | 440.11 (47.38) | -0.05 | 440.44 (45.43) | 435.78 (44.91) | 0.36 |

*Note.* M = Mean, SD = Standard Deviation.

# Supplementary Figures

## Supplementary Figure 1

*Experiment 1 Grand Averaged ERPs Across All Electrodes by Task Condition and Word Type*

*
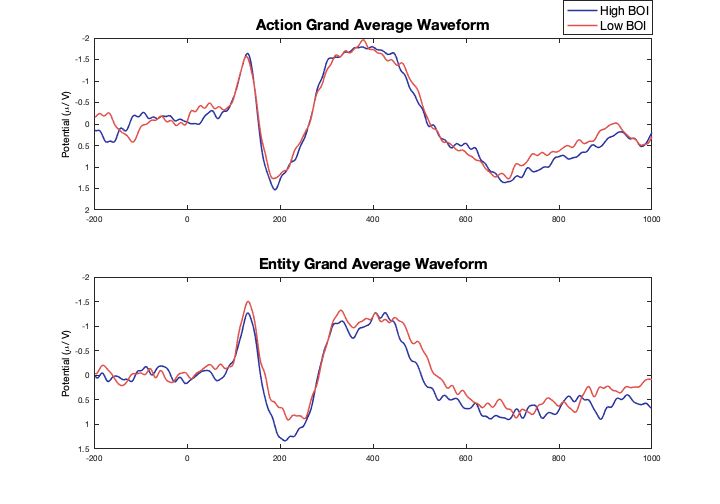
*

*Note.* Grand average was calculated from a pre-defined central cluster of electrodes (FC1, FCz, FC2, C1, Cz, C2, CP1, CPz, CP2).

## Supplementary Figure 2

*Experiment 1 Grand Averaged, electrode-specific ERPs by Task Condition and Word Type*


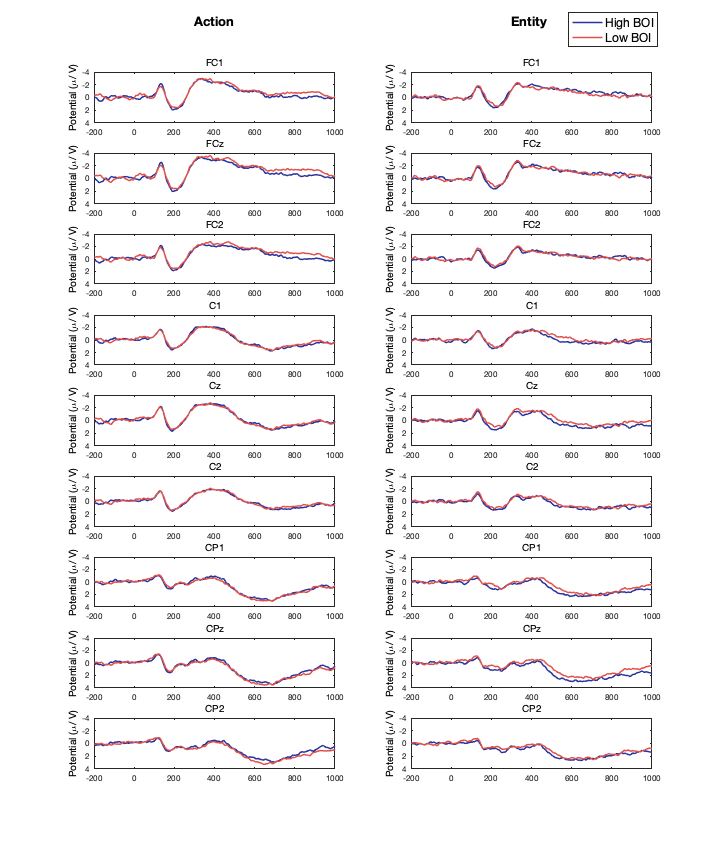

Supplement: Supplementary file 1 [file Data_Sheet_1.docx]
